# Adrenocorticotropic hormone therapy for the treatment of idiopathic nephrotic syndrome in children and young adults: a systematic review of early clinical studies with contemporary relevance

**DOI:** 10.1007/s40620-016-0308-3

**Published:** 2016-04-16

**Authors:** Kenneth V. Lieberman, Anna Pavlova-Wolf

**Affiliations:** 1 Hackensack University Medical Center, 30 Prospect Avenue, Hackensack, NJ 07601 USA; 2 Mallinckrodt Pharmaceuticals, Hayward, CA USA

**Keywords:** Adrenocorticotropic hormone, Nephrotic syndrome, Pediatric, Systematic review

## Abstract

Adrenocorticotropic hormone (ACTH) as a treatment for proteinuria due to nephrotic syndrome (NS) has re-emerged over the last decade. Current clinical data are primarily limited to adults with treatment-resistant NS. Largely unknown to today’s clinicians is the existence of early clinical studies, following ACTH’s introduction in the late 1940s, showing sustained proteinuria response in idiopathic NS in predominantly pediatric, treatment-naïve patients. Before ACTH, patients suffered severe edema and high mortality rates with no reliable or safe treatment. ACTH dramatically altered NS management, initially through recognition of diuresis effects and then through sustained proteinuria remission. This review synthesizes early clinical literature to inform current NS patient management. We undertook a MEDLINE search using MeSH terms “adrenocorticotropic hormone” and “nephrotic syndrome,” with limits 1945–1965 and English. Sixty papers totaling 1137 patients were found; 14 studies (9 short-term, five long-term, N = 419 patients) met inclusion criteria. Studies were divided into two groups: short-term (≤28 days) and long-term (>5 weeks; short-term initial daily treatment followed by long-term intermittent)ACTH therapy and results were aggregated. An initial response, defined as a diuresis, occurred in 74 % of patients/treatment courses across nine short-term ACTH studies. Analyzed in eight of these studies, proteinuria response occurred in 56 % of patients/treatment courses. Across five long-term ACTH studies, proteinuria response was shown in 71 % of patients and was sustained up to 4.7 years following treatment. The inventory and re-evaluation of early clinical data broadens the evidence base of clinical experiences with ACTH for implementation of current treatment strategies and aiding the design of future studies.

**Electronic supplementary material:**

The online version of this article (doi:10.1007/s40620-016-0308-3) contains supplementary material, which is available to authorized users.

## Introduction

Adrenocorticotropic hormone (ACTH) re-emerged over the last decade as a treatment for nephrotic syndrome (NS) following studies in Europe and the United States. Proteinuria reduction was shown using a synthetic ACTH intramuscular depot formulation (tetracosactide), available in Europe but not the US [1, 2]. Later, a randomized, controlled trial compared treatment with this compound administered twice weekly for 1 year to alternating months of methylprednisolone and cyclophosphamide or chlorambucil for 6 months (the Ponticelli regimen). This study showed comparable high remission rates in patients with idiopathic membranous nephropathy with both treatments [3]. More recently, the pituitary extract-derived compound H.P. Acthar^®^ Gel (repository corticotropin injection; Mallinckrodt ARD Inc., Hazelwood, MO) has significantly reduced proteinuria in some patients with NS with a variety of histologic patterns [4–9]. These reports studied relatively small, heterogeneous patient groups for limited time periods and most patients had treatment-resistant NS prior to initiation of Acthar Gel. Almost all the subjects were adults with either membranous nephropathy or focal segmental glomerulosclerosis. Thus, current clinical data examining ACTH treatment of NS are very limited, especially in pediatric patients.

The early ACTH clinical studies are largely unknown to today’s practicing clinician. Yet, the majority of clinical studies that evaluated ACTH treatment for NS were conducted in the 1950s using a compound purified from pituitary extract. These early studies evaluated predominantly pediatric, treatment-naïve patients with idiopathic NS who likely would have been diagnosed with minimal change disease (MCD). Prior to the emergence of ACTH in the late 1940s, there was no reliable treatment that provided symptom relief or remission. Patients suffered from generalized, severe edema that could remain for months, often resulting in semi-incapacity to complete disability. They were extremely vulnerable to infection and the mortality rate was as high as 40–71 % within approximately 4–5 years of NS onset [10–12]. Treatments, such as intentional infection with measles or malaria, infusion of concentrated serum albumin, nitrogen mustard and mercurial diuretics yielded variable results and were associated with significant risks [13–16].

The introduction of ACTH (purified from a pituitary extract) dramatically altered NS management, initially most visibly by providing relief from gross edema through diuresis and then with longer treatment courses enabling sustained proteinuria remission [14, 17]. Prolonged, intensive ACTH or steroid therapy improved survival compared to historical control patients; the mortality rate declined to approximately 20–23 % [11, 12, 18]. The initial ACTH investigations across a broad range of diseases, including NS, are described in the Proceedings of the First Clinical ACTH Conference, held in 1949 in Chicago [19, 20]. Studies examining ACTH therapy for NS were published throughout the 1950s, including case studies and large patient cohorts [10, 13–17, 21–40]. ACTH was largely replaced with the synthetic oral steroid prednisone in the 1960s due to its greater ease of administration and more reliable availability [10]. No comparative effectiveness clinical trials were ever conducted. Additionally, at that time the possible differing mechanisms of action between ACTH and steroids or that ACTH may have steroid-independent pathways were not understood. Treatment of infantile spasms (IS) was an exception to the shift toward steroid treatment. Neurologists continued to use ACTH as a primary treatment for IS since the initial 1958 report by Sorel and Dusaucy-Bauloye [41].

A critical need remains for effective treatment for patients with NS who are not responsive to the standard regimen of orally administered steroid without the renal and extra-renal toxicities often associated with chronic use of current therapies [42]. Analysis of the early clinical studies addresses an important need in expanding clinical experience and understanding ACTH treatment in NS. The aim of the current retrospective review and analysis is to broaden the evidence base underlying implementation of current ACTH therapy and aid in the design of future robust studies. The current systematic review examines the largest patient population receiving ACTH treatment available to date and examines three periods within the clinical development of ACTH treatment. First, the effects of short-term, daily ACTH on diuresis with edema resolution, the clinical response focus when ACTH was first introduced, were examined. This initial treatment response led early investigators to hypothesize that ACTH was a diuretic. Second, following the realization that ACTH had an important treatment role beyond diuresis, the focus shifted to the effects of short-term ACTH on proteinuria. Third, studies evaluated the effects of long-term intermittent ACTH therapy on sustained proteinuria response.

## Methods

**Fig. 1:**
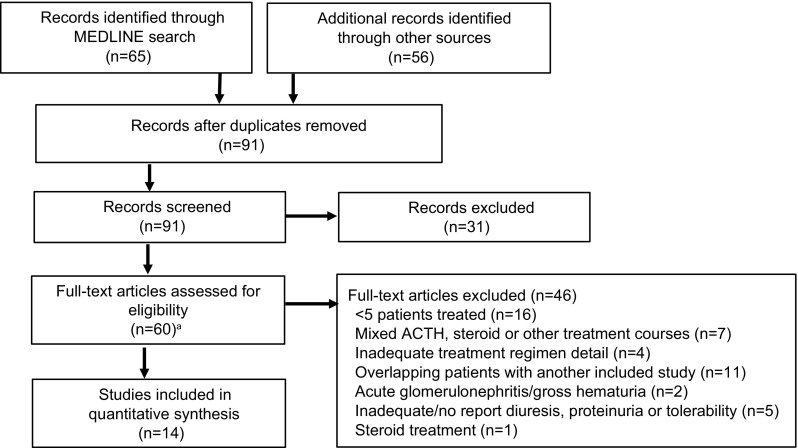
Identification and selection of early clinical ACTH studies. ^a^The complete list of the 60 articles assessed for eligibility is available in the on-line table. *ACTH* adrenocorticotropic hormone

MEDLINE was searched using the MeSH terms “adrenocorticotropic hormone” and “nephrotic syndrome,” with the limits 1945 (era of ACTH introduction) to 1965 (ACTH was largely replaced by synthetic oral steroids by this time) and English. Studies also were selected from reference lists of reviewed papers and author K.V.L.’s historical collection. Study selection is shown in Fig. 1. In studies that examined ACTH and steroid treatment, only ACTH-related data were considered. Studies that focused on acute glomerulonephritis with gross hematuria were excluded. When multiple papers used overlapping patient groups, only one was included.

Inclusion criteria included: >5 patients treated; reporting on the clinical response of diuresis and edema resolution; proteinuria (defined quantitatively or semi-quantitatively); and tolerability. Outcomes were tabulated using counts and percentages. Seven of the nine short-term studies and all five long-term studies reported treatment outcomes by number of patients. Two short-term studies reported outcomes by number of treatment courses. The aggregation of proteinuria response and diuresis data among short-term studies combined the number of patients from seven studies and the number of treatment courses from ywo studies. Tolerability was characterized descriptively. Short-term treatments were defined as daily ACTH for ≤4 weeks. Long-term treatments had an initial daily induction regimen followed by intermittent dosing schedules for ≥5 weeks beyond the initial treatment course.

## Results

Sixty papers with 1137 patients were identified (on-line Table). Inclusion/exclusion criteria were met by 14 studies that included 419 patients. Nine studies with 330 patients examined clinical responses (diuresis with edema resolution) to short-term ACTH. Eight of these studies included data on proteinuria response in 225 patients. Five studies with 89 patients reported sustained proteinuria response with long-term treatment. The patients were predominantly pediatric (88 %; 341/388 with ages given) with age ≤20 years [10, 13, 14, 17, 31–40]. Report of proteinuria response was semi-quantitative, including marked reduction, absence or trace level, within normal limits and 1+ level.

### Short-term ACTH therapy

Short-term ACTH treatment results for diuresis response and proteinuria are summarized in Table 1 [10, 14, 31–37]. ACTH dose ranged from 20 mg/day to 160 mg/day, with one protocol using the regimen of 150–200 mg/m^2^/day [35]. Mean treatment duration for a single treatment course was approximately 12 days, ranging 4–28 days. Patients who relapsed following cessation of short-term treatment or who did not respond adequately often received additional short-term ACTH courses [10, 14, 31–36].

**Table 1:** Proteinuria and diuresis response during short-term ACTH therapy for nephrotic syndrome

Study	N	Age (n)	ACTH regimen Dose and duration	Diuresis response N (%)	Proteinuria response N (%)	Response duration N (%)
Luetscher [34]	14	≤10 (7) 16 (1) ≥22 (6)	25–100 mg/d 9–14 d	10 (71)	11/14 (79) response 4/14 (29) absence	3/10 (30) up to 10 mo^i^ 7/10 (70) ≤4 mo^i^
Rapoport [36]	34	<8 (34)	40–150 mg/d 8–12 d	28 (82)	12/12^e^ (100) response	13/28 (72) up to 18 mo^i^
Kramer [14]	12	≤18 (12)	50–100 mg/d up to 125 mg/d 8–13 d	9 (75)	9/12 (75) absence/trace	1/12 (8) 12 mo^j^ 7/12 (58) 1–9 mo^j^
Metcoff [35]	45	<12 (45)	150–200 mg/m^2^/d 8–10 d	34 (75)	3/12^f^ (25) response	NA
Riley [37]	50	<18 (49)	60 mg/d (age < 4) 80 mg/d (age 4–9) 100 mg/d (age > 9) 10 d	41/63^c^ (65)	NA	111/140 (79) ≤3 mo^i,k^
Rance [10]	88	≤18 (88)	20–50 mg/d^b^ 4–28 d	75/109^c^ (69)	44/109^g^ (40) absence 25/33^h^ (76) absence	NA
Baskin [31]	18	≤18 (18)	50–100 U/d 7–14 d	15 (83)	17/18 (94) response 7/18 (41) absence	NA
Charlton [32]	38	≤10 (21) 11–20 (8) ≥21 (9)	20–160 mg/d 4–23 d	25/30^d^ (83)	22/38 (58) absence	12/22 (54) ≥12 mo^j^
Eales [33]	31	Range <10 to ≥61^a^	100 mg/d^b^ 7–20 d	28 (90)	13/31 (42) response	4/31 (13) >2 yr^j^
Total patients showing response N (%)				265/356 (74)	156/279 (56)	

*ACTH* adrenocorticotropic hormone, *d* day(s), *mo* month(s), *NA* not available, *yr* year(s)

^a^Age was not specified for the subset of patients who received ACTH treatment

^b^1 or more patients received one or more courses of ACTH treatment by intravenous infusion

^c^Percentage of ACTH treatment courses

^d^30/38 patients presented with edema

^e^12/34 patients had assessment of proteinuria

^f^12/45 patients completed proteinuria assessment pre- and post-ACTH therapy

^g^Number of courses with edema present pre-treatment

^h^Number of courses without edema at pre-treatment

^i^Diuresis with edema resolution response duration

^j^Proteinuria response duration

^k^Treatment courses included ACTH and/or cortisone

Diuresis with edema resolution was the key clinical outcome in the earliest studies of short-term ACTH and ACTH dose was quickly tapered or discontinued following diuresis onset [14, 37]. The nine short-term ACTH treatment studies reported diuresis with edema resolution in 74 % (265/356) of patients/treatment courses [14, 31–36]. Compared with the six short-term ACTH studies showing diuresis in ≥75 % of patients, the three studies that reported diuresis in <75 % of patients had shorter treatment duration, courses of 4–6 days, or lower ACTH dosage, 20–25 mg/day (Table 1). Onset of diuresis occurred either during or within a few days following the cessation of ACTH treatment. Diuresis was usually temporary, with the majority of patients relapsing within 1 month [33].

The eight short-term ACTH treatment studies that reported proteinuria showed a proteinuria response in 56 % (156/279) of patients/treatment courses. Using the more restrictive definition of proteinuria absence, 50 % (139/279) of patients/treatment courses showed a proteinuria response. The duration of treatment effects on proteinuria were described as transient for the majority of patients, lasting a few days to a few months following ACTH cessation, or were not examined [10, 14, 31, 33–35]. A study at the upper end for treatment duration (up to 21 days) and ACTH dose (160 mg/day) found 12/22 (54 %) patients who had shown proteinuria absence following ≥1 courses of ACTH had sustained proteinuria remission at their ≥12 month post-treatment assessment [32].

### Long-term ACTH therapy

Long-term ACTH treatment results are summarized in Table 2 [13, 17, 38–40]. All regimens included an initial short-term daily ACTH treatment course. The ACTH intermittent regimen doses ranged from 100 to 200 mg/day, with the exception of one protocol that used 1 mg/lb/day [40]. The regimens of four studies included intermittent ACTH treatment for three consecutive days repeated weekly for up to 24 months. One study used long-term daily ACTH treatment for up to 19 months as maintenance therapy [38]. In the remaining study, the initial ACTH treatment course was followed by daily ACTH treatment at 1 mg/lb/day until absence of albuminuria for 1–2 weeks [40]. Treatment then shifted to an intermittent, every-other-day regimen with ACTH increased to 1.2 mg/lb/day if no response was shown by 3 weeks, followed by reduction in ACTH dose over several weeks. In this treatment protocol, if albuminuria increased during the intermittent regimen, the dose or frequency of ACTH was doubled or increased to the initial treatment dose.

**Table 2:** Proteinuria response to long-term ACTH therapy for nephrotic syndrome

Study	N	Age (n)	Initial course and maintenance ACTH regimens Dose and duration	Proteinuria response during ACTH treatment N (%)/duration	Proteinuria response Post-ACTH treatment N (%)/duration
Lange [39]	6	≤18 (6)	*Initial course* 100 mg/d for 7 d *Maintenance therapy* 100 mg/d for three consecutive d, repeated weekly for 5–8 wk	6/6 (100)/5–8 wk	5/6 (83)/6–26 mo
Merrill [40]	25	≤12 (25)	*Initial course* 1 mg/lb/d for 10 d *Maintenance therapy* 1 mg/lb/d until stable response, continued 2 wk to 3 mo If no response after 3 wk, ACTH dose increased, given every other day, continued 2 wk to 17 mo Minimum 4 mo maintenance treatment with decreasing dose tapered to 1 mg/lb/d twice weekly for several wk If relapse (defined as 2+ to 4+ albuminuria), dose or frequency doubled If proteinuria not diminished within 48 h of relapse, ACTH dose increased to initial level	23/25 (92)/≤25 mo	14/25 (56)/1 wk–18 mo
Durand [13]	11	≤18 (11)	*Initial course* 100 mg/d age ≤ 6; 150–200 mg/d age >6 average 8 d *Maintenance therapy* Initial course regimen repeated with 3- to 4-d no-treatment intervals “until stable response”	11/11 (100)/NA	NA
Mateer [17]	9	≤13 (9)	*Initial course* 100–200 mg/d for 28 d *Maintenance therapy* 100 mg, three times per week, repeated weekly	9/9 (100)/≥ 12 mo^a^	NA^a^
Danowski [38]	38	≤20 (7) >20 to ≤69 (31)	*Initial course* 50–500 U daily, typically 200 U/d for ≥28 d *Maintenance therapy* 200 U daily for three consecutive d, repeated weekly for 2 to 24 mo or daily ACTH up to 19 mo	14/38 (37)/1 mo to 4.7 yr^a^	NA^a^
Total patients showing proteinuria response, N (%)				63/89 (71)	19/31 (61)
Total patients duration of treatment/follow-up, range				1 mo–4.7 yr	

*ACTH* adrenocorticotropic hormone, *d* day(s), *lb* pounds, *mo* month(s), *NA* not available, *yr* year(s)

^a^Insufficient study detail to determine the period on long-term ACTH treatment versus post-treatment during sustained proteinuria

Sustained proteinuria response was examined in the five long-term studies. Evaluation of proteinuria response during long-term ACTH or combined long-term ACTH and post-treatment follow-up showed proteinuria response in 71 % (63/89) of patients [13, 17, 38, 40]. The two studies that reported proteinuria response post-treatment showed 61 % (19/31) of patients had sustained proteinuria response for up to 26 months [39, 40].

Patient follow-up ranged from 1 month to 4.7 years [13, 17, 38–40]. In a study of 25 patients, sustained proteinuria response following ACTH cessation was shown in 14 patients for a mean of 9.6 months and another nine patients showed continued proteinuria response during their ongoing ACTH intermittent regimen [40]. A study of 38 patients, primarily adults, found proteinuria response in 18 (47 %) patients following the initial course of ACTH. Twelve (67 %) of these patients showed sustained proteinuria response during the long-term ACTH regimen or post-ACTH treatment for up to 4.7 years [38]. An additional two patients who did not show proteinuria response following the initial ACTH course did show proteinuria response after long-term (13 and 14 months) intermittent ACTH treatment at the 1- and 3-year evaluations. Following 5–8 weeks of an intermittent ACTH regimen, 5/6 (83 %) patients showed sustained proteinuria response at 6–26 months following ACTH cessation [39]. Two studies reported 9/9 and 11/11 patients showed sustained proteinuria response following long-term ACTH without providing the specific treatment duration versus post-treatment assessment [13, 17].

## Tolerability

The early clinical studies noted a worsening of edema at initiation of ACTH therapy, prior to diuresis [32–34]. Although not yet well known at the time, we can now conclude that the worsening of edema was likely due to fluid retaining effects via the steroidogenic effects of ACTH. Side effects associated with hyperadrenocorticism, including characteristics of Cushing’s syndrome, were described during short-term, high-dose (ranging from 100 to 160 mg/day) daily ACTH treatment, but resolved following ACTH cessation [14, 32, 33, 36]. Additionally, freckling was reported in nearly all patients in one study [17]. Long-term intermittent ACTH treatment studies described less pronounced hyperadrenocorticism side effects [17, 38, 40].

## Discussion

ACTH transformed the treatment of patients with NS in the late 1940s through the 1950s. There was a dramatic drop in mortality from as high as 40–71 % before the introduction of ACTH to 23 to 25 % by 1958 [11, 12, 18]. Following the first clinical use of ACTH in NS by Edith Farnsworth in 1948, short-term ACTH regimens with daily high-dose treatment targeted at diuresis were introduced to bring relief and improve quality of life in greatly suffering edematous patients [19, 20].

In the current examination of the early clinical literature, 74 % of patients achieved a diuresis in response to one or more short-term ACTH courses. However, proteinuria completely resolved in only 54 % of the historical patients who received short-term treatment. This lower response rate as compared with current regimens is likely due to the briefer treatment course. The brevity of the diuresis and the variable effect on proteinuria led some early investigators to consider ACTH a diuretic without effect on the core pathophysiologic alterations of NS. A report from the International Study of Kidney Diseases in Children (ISKDC) described time to response (here defined as proteinuria resolution) for patients with MCD treated with prednisone [43]. A similar response rate was seen in patients treated with prednisone for 1–2 weeks as was seen in the short-term ACTH experience. In the short-term ACTH studies, a dose–response association was recognized. Examination of patient outcomes relative to treatment duration showed that treatment <8 days was ineffective whereas 83 % of patients showed loss of edema, proteinuria or both when treatment was ≥18 days [10]. Similarly, it was stated that the proteinuria response was more pronounced with 4 weeks as compared to 1–2 weeks of treatment [17].

A key goal of long-term ACTH treatment was to induce a proteinuria response in patients who had not responded to short-term therapy, in modern terms to overcome steroid resistance, and the long-term studies showed a greater percentage of patients achieving proteinuria remission [38]. These early findings are consistent with modern treatment regimens using longer-term administration schedules. Some patients who were resistant when treated short-term then responded to a longer treatment regimen. That the proteinuria response rate of 54 % in short-term studies improved to 71 % during long-term treatment supports an important modern treatment indication for ACTH in patients who are steroid resistant.

The transient proteinuria response during the early short-term clinical trials also led some clinical researchers to consider whether longer ACTH treatment could modify the course of NS and produce a sustained proteinuria remission [31]. ACTH regimens evolved to longer duration of continuous treatment; repeated intermittent treatment cycles with short intervals of no treatment; and the inclusion of additional ACTH courses in patients who had already achieved a diuresis [13, 32, 44–46]. Barnett and colleagues hypothesized in 1954 that proteinuria in patients with NS was due to increased permeability of the glomerular capillary wall to proteins such as albumin [21]. Following an ACTH treatment study in 10 children with NS in which glomerular filtration was also serially evaluated, Barnett and colleagues concluded that the primary effect of ACTH treatment is the shift toward normal of glomerular permeability to albumin and other plasma proteins.

Long-term intermittent regimens treated patients with ACTH from 5 weeks to 2 years beyond the initial short-term daily ACTH regimen with follow-up of patient outcomes up to 4 years post-ACTH therapy [13, 17, 38–40]. In the current examination of the early clinical studies, the long-term study that primarily examined adults showed 37 % were maintained in long-term remissions during ACTH treatment and/or post-treatment up to 4.7 years [38]. Among the four long-term studies that included children, ≥92 % of patients were maintained in remissions during ACTH treatment [13, 17, 39, 40]. In one study, 83 % of patients showed sustained proteinuria response up to 26 months post-treatment and in another study, 56 % of patients showed sustained proteinuria response up to 18 months post-treatment [39, 40]. The high rate of sustained remissions in children receiving longer duration formulation ACTH treatment supports the use of ACTH therapy in patients who are steroid dependent (SD) or frequently relapsing (FR), two important modern treatment needs.

The multinational, collaborative work of the ISKDC during the 1970s resulted in histopathologic classification (utilizing the newly introduced technique of percutaneous kidney biopsy) and the clinical characterization system based on steroid response. The current review found 71 % of patients achieved proteinuria response with long-term intermittent ACTH treatment, which is consistent with later reports of response rates with longer-term steroid treatment [43]. Patients included in long-term ACTH treatment to maintain proteinuria remission would fit later definitions of SD, steroid resistant (SR) and FR. In one long-term study, patients who experienced ≥1 exacerbations were placed on intermittent or continuous ACTH therapy [17]. Among 34 exacerbations, 18 patients were re-treated 1–6 times and the proteinuria response to retreatment with ACTH was comparable to the initial treatment response [17]. In another long-term study, continuous treatment was initiated in patients who relapsed following the initial treatment course [40]. Among the 12 patients, there was an average of four failed intermittent treatment courses before continuous treatment was implemented, resulting in sustained remissions [40].

Different ACTH formulations, and purification and standardization protocols during the 1950s (with the potential for impurities) likely led to more variability in patient outcomes and side effects than would be expected with the currently available, FDA approved, highly purified Acthar Gel formulation. Of the eight studies that reported their ACTH source, four named Armour Laboratories (a precursor of Acthar HP gel in the literature) [34–36, 39], two stated the National Drug Company [17, 38], and one each identified the Medical Research Council [32] and Connaught Laboratories/Nordic Biochemicals Limited [10]. Comparison of dosages is also problematic. In some cases, the active ingredient was quantified in mg or mcg/mL, whereas in other products it was characterized by biologic potency or activity in U/mL. Acthar Gel is approved by the FDA based on potency (U/mL). Although it is possible to qualitatively compare efficacy of Acthar Gel (dosed in U/mL) with that of historical ACTH preparations (dosed in mg or mcg/mL) in a given indication, it is not possible to quantitatively compare the specific dosing regimens.

These early clinical studies focused primarily on children. The most common diagnosis in current nosology was likely MCD, as indicated by the high percent of prompt remissions that were observed. Focal segmental glomerulosclerosis was probably present in some patients with short-term treatment resistance. Current clinical studies examining ACTH treatment in children with NS are scarce, yet the early clinical literature suggests ACTH may be very beneficial for these patients [47]. Additional effective, tolerable treatment options are of particular need among children with NS who are nonresponsive to or unable to tolerate steroid or other first- or second-line treatments.

A central question for today’s clinicians is which patient subgroups respond to ACTH treatment and what underlies differential responsiveness. One possibility relates to the total cumulative ACTH dose needed for a clinical response and the balance between cumulative dose, treatment duration, and potential side effects in patient subgroups. For example, among patients with idiopathic membranous nephropathy, those receiving greater cumulative dose ACTH gel (2800 U) showed greater proteinuria reduction compared with lower cumulative doses (880 and 1760 U) [7]. Although the early clinical studies did not provide sufficient details to determine cumulative dose, the differences between short-term daily and long-term intermittent ACTH regimens in producing sustained proteinuria response supports that such a balance is important for optimal patient outcomes.

**Fig. 2:**
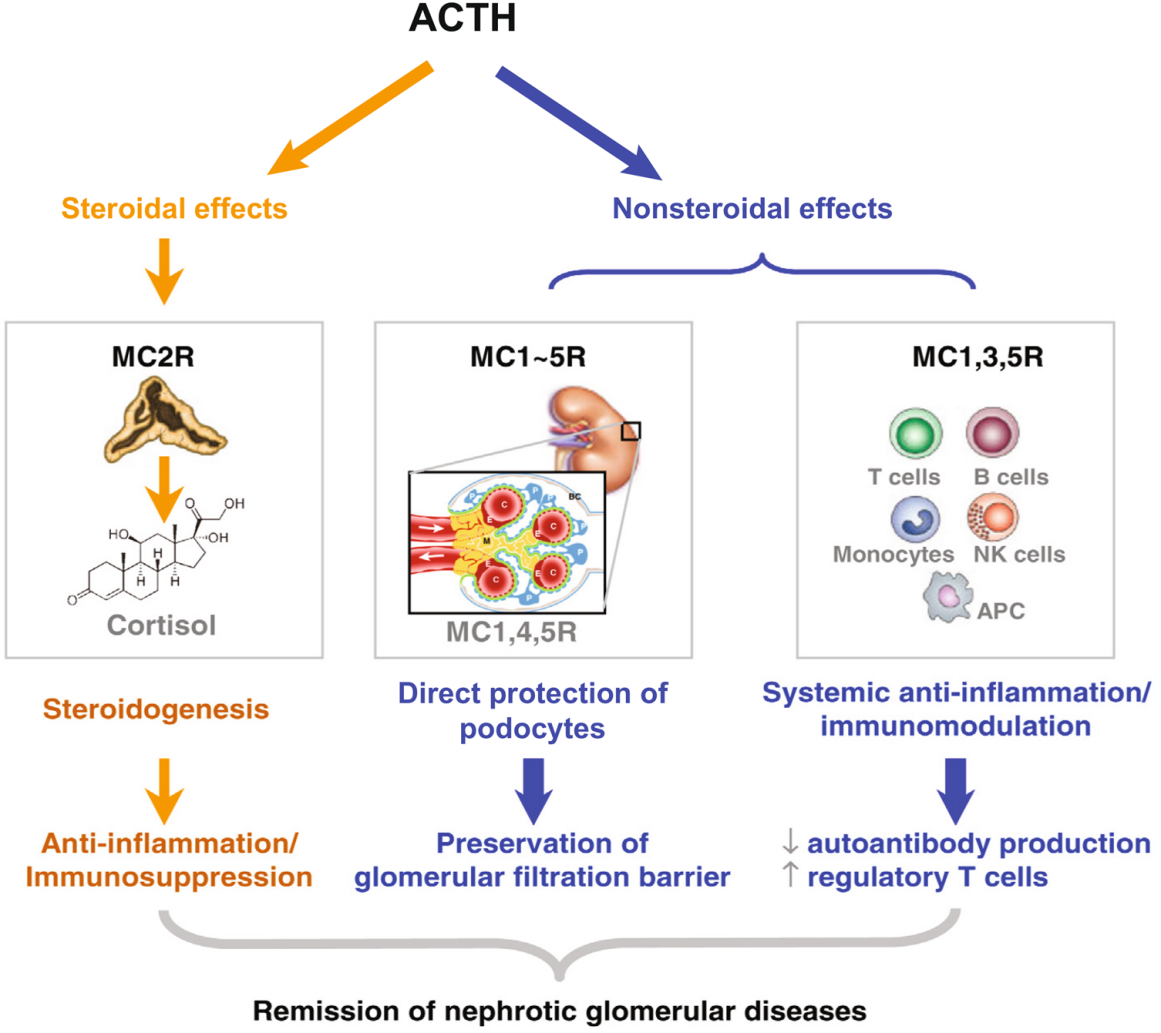
Putative mechanisms of action of ACTH in the kidney. *ACTH* adrenocorticotropic hormone, *APC* antigen-presenting cells, *MC* melanocyte, *NK* natural killer, *R* receptor Adapted from Ref. [49], page 141, Copyright 2014, with permission from Elsevier

One early clinical study noted freckling, a probable melanocyte effect, in nearly all patients, an early indication of a possible non-steroid pathway of action for ACTH [17]. A recent focus in understanding patient responsiveness to ACTH therapy in NS is the mechanism of action of ACTH through the interaction of ACTH with melanocortin receptors (MCRs) and their steroid dependent and independent pathways [48–50]. Steroid-independent effects may occur through MC1R [51, 52]. ACTH in an animal model of progressive renal tubulointerstitial injury has shown suppression of tubulointerstitial inflammation, tubular atrophy, and fibrosis through anti-inflammatory effects mediated by MC1R on tubular epithelial cells [51]. MC1Rs have been shown in podocytes, glomerular endothelial cells, and mesangial cells, and an MC1R agonist resulted in significantly reduced proteinuria in the passive Heymann nephritis animal model [52]. Such steroid-independent mechanisms of action may provide an explanation for ACTH efficacy in steroid-resistant patients (Fig. 2).

The current systematic review has several limitations. Indexing of published studies was not exhaustive during the 1950s, resulting in some papers being unavailable for review. Some papers used a discussion format and did not provide comprehensive clinical information. Modern nomenclature was not available for patient diagnoses and categorization as there was no clinical classification, such as “steroid responsive,” or histological classifications as are used today. Despite the absence of modern terminology, the detailed clinical descriptions in the selected manuscripts enables a useful analysis of this experience. Potential risk of bias in the publication of early clinical studies could not be formally assessed.

## Conclusion

The potential of ACTH to produce sustained proteinuria response in idiopathic NS was shown at its introduction in the 1950s. This historical experience, which provides the largest population of patients with NS treated with ACTH to date and broadens the evidence base of clinical experience with ACTH, has the following contemporary implications. Daily ACTH can be effectively substituted for daily prednisone for the initial treatment of newly presenting patients with NS as well as for the treatment of relapses in patients already known to be steroid responsive. This approach with ACTH treatment has already been shown to be very useful for patients who are not able to tolerate oral steroid treatment [47]. Patients with chronic gastritis, for whom oral steroid administration is relatively contraindicated, could also benefit from the parenteral administration of ACTH. Determining whether this regimen would result in a higher percentage of treatment responses or more sustained remissions compared with the current standard regimen will require an appropriately powered clinical trial.

The historical data suggest a role for ACTH therapy for the more problematic patients with SD, FR and SR patterns. Longer-term intermittent ACTH courses were well tolerated and induced remissions at a higher rate than shorter regimens as well as sustained long-term remissions for many patients. With current regimens, these patients suffer significant cumulative steroid toxicity and are often exposed to the toxicities of intensive immunosuppressive therapies. A clinical trial of ACTH treatment of SD and FR patients is currently recruiting and a clinical trial for the treatment of SR patients is in the final stage of planning. Demonstration of ACTH responsiveness in steroid-resistant children (as demonstrated in adults) would represent a significant advance in therapeutics [4–7].

## Electronic supplementary material

Below is the link to the electronic supplementary material.

Supplementary material 1 (DOCX 36 kb)
