# Supplementary material for: Adrenocorticotropic hormone therapy for the treatment of idiopathic nephrotic syndrome in children and young adults: a systematic review of early clinical studies with contemporary relevance
Source: J Nephrol. 2016 Apr 16;30(1):35–44. doi: 10.1007/s40620-016-0308-3 (PMC5316399; doi:10.1007/s40620-016-0308-3)
Supplement: Supplementary file 1 — Supplementary material 1 (DOCX 36 kb) [file 40620_2016_308_MOESM1_ESM.docx]

**Online Supplementary Table to accompany:**

**Article title:** Adrenocorticotropic Hormone Therapy for the Treatment of Idiopathic Nephrotic Syndrome in Children and Young Adults: A Systematic Review of Early Clinical Studies with Contemporary Relevance

**Submitted to:** *Journal of Nephrology*

**Authors:** Kenneth V. Lieberman, MD^1^, and Anna Pavlova-Wolf, PhD^2^

**Affiliations:** ^1^Hackensack University Medical Center, Hackensack, NJ; ^2^Mallinckrodt Pharmaceuticals, Hayward, CA

**Corresponding author email address:** klieberman@hackensackUMC.org

**ACTH Treatment of Patients With Nephrotic Syndrome From 1950 to 1965**

| Study | Unique Patients Treated With ACTH  N |
| --- | --- |
| Aber 1954^1^ | 2 |
| Barnett 1950^2^ | 0^a^ |
| Barnett 1951^3^ | 0^a^ |
| Barnett 1952^4^ | 0^a^ |
| Baskin 1956^5^ | 18 |
| Bjorneboe 1952^6^ | 7 |
| Brown 1965^7^ | 0^a^ |
| Burke 1958^8^ | 58 |
| Charlton 1958^9^ | 40 |
| Danowski 1957^10^ | 0^a^ |
| Danowski 1959^11^ | 54 |
| Dundon 1957^12^ | 8 |
| Durand 1955^13^ | 16 |
| Eales 1959^14^ | 31 |
| Eiben 1954^15^ | 1 |
| Farnsworth 1950^16^ | 8 |
| Farnsworth 1950^17^ | 3 |
| Farnsworth 1950^18^ | 7 |
| Frankel 1950^19^ | 1 |
| Fujiwara 1962^20^ | 1 |
| Greenman 1955^21^ | 30 |
| Hecker 1952^22^ | 1 |
| Heidorn 1955^23^ | 7 |
| Heidorn 1956^24^ | 0^a^ |
| Heidorn 1955^25^ | 10 |
| Heymann 1955^26^ | 64 |
| Heymann 1955^27^ | 10 |
| Hooft 1959^28^ | 22 |
| Hooft 1959^29^ | 1 |
| Kohn 1960^30^ | 6 |
| Kramer 1952^31^ | 12 |
| Kramer 1952^32^ | 8 |
| Lange 1953^33^ | 16 |
| Lange 1955^34^ | 18 |
| Lange 1957^35^ | 0^a^ |
| Lange 1958^36^ | 12 |
| Lausen 1952^37^ | 8 |
| Lausen 1954^38^ | 2 |
| Leyva 1953^39^ | 20 |
| Luetscher 1951^40^ | 14 |
| Mateer 1957^41^ | 42 |
| McCall 1952^42^ | 3 |
| McKean 1953^43^ | 1 |
| Merrill 1954^44^ | 25 |
| Metcoff 1952^45^ | 45 |
| Michaels 1953^46^ | 2 |
| Rance 1955^47^ | 88 |
| Rapoport 1950^48^ | 0^a^ |
| Rapoport 1951^49^ | 34 |
| Riley 1952^50^ | 50 |
| Riley 1956^51^ | 260^b^ |
| Rinvik 1952^52^ | 2 |
| Schemm 1957^53^ | 12 |
| Schoolman 1956^54^ | 1 |
| Shreeve 1955^55^ | 1 |
| Singh 1952^56^ | 1 |
| Soffer 1954^57^ | 32 |
| Soshea 1951^58^ | 6 |
| Stickler 1956^59^ | 4 |
| West 1958^60^ | 12 |

^a^Paper discussed patients who were presented in another publication included within the table.

^b^Estimated proportion of patients receiving ACTH among 780 patients receiving either ACTH, cortisone, or hydrocortisone.

**References**

1. Aber GM, Chandler GN, Hartfall SJ. Cortisone and A.C.T.H. in treatment of non-rheumatic conditions. *Br Med J.* 1954;1(4852):1-8.

2. Barnett HL, McNamara H, McCRORY W, et al. The effects of ACTH and cortisone on the nephrotic syndrome. *AMA Am J Dis Child.* 1950;80(3):519-520.

3. Barnett HL, Forman CW, McNamara H, McCory WW. The effect of adrenocorticotrophic hormone on children with the nephrotic syndrome. II. Physiologic observations on discrete kidney functions and plasma volume. *J Clin Investig.* 1951;30(2):227-235.

4. Barnett HL. Effect of ACTH in children with the nephrotic syndrome. *Pediatrics.* 1952;9(3):341.

5. Baskin JL. Medical management of renal disease in infants and children. *Tex State J Med.* 1956;52(12):865-869.

6. Bjorneboe M, Brun C, Gormsen H, Iversen P, Raaschou F. The nephrotic syndrome. II. The effect of corticotropin ACTH. *Acta Med Scand.* 1952;142(suppl 266):249-265.

7. Brown RB, Burke EC, Stickler GB. Studies in nephrotic syndrome. I. Survival of 135 children with nephrotic syndrome treated with adrenal steroids. *Mayo Clin Proc.* 1965;40:384-390.

8. Burke EC. Survival in a group of steroid-resistant nephrotic children; preliminary report. *Proc Staff Meet Mayo Clin.* 1958;33(1):12-18.

9. Charlton D, Latner AL, Platt JW, Smart GA, Thompson RB, Walker W. The nephrotic syndrome. Observations of the effects of A.C.T.H. in 40 patients. *Acta Med Scand.* 1958;161(1):33-56.

10. Danowski TS, Weigand FA, Greenman L, Gailani S, Greenberg WV, Mateer FM. Corticotropin (ACTH) therapy of nephrotic syndrome in children. II. Laboratory findings in one hundred six instances. *AMA J Dis Child.* 1957;93(6):604-614.

11. Danowski TS, Mateer FM, Puntereri AJ. ACTH or adrenocortical steroid therapy of proteinuria in adolescents and in adults. *Am J Med Sci.* 1959;237(5):545-558.

12. Dundon S. Steroid therapy in the nephrotic syndrome. *J Ir Med Assoc.* 1957;40(239):135-144.

13. Durand P, DeToni E, Jr. Treatment of nephrotic syndrome in children. *Ann Paediatr.* 1955;185(4):225-235.

14. Eales L. The effect of ACTH and the steroid drugs on the nephrotic syndrome. *S Afr J Lab Clin Med.* 1959;5:125-155.

15. Eiben RM, Kleinerman J, Cline JC. Nephrotic syndrome in a neonatal premature infant; report of a case. *J Pediatr.* 1954;44(2):195-202.

16. Farnsworth EB. Metabolic changes associated with administration of adrenocorticotropin in the nephrotic syndrome. *Proc Soc Exp Biol Med.* 1950;74(1):60-62.

17. Farnsworth EB. Acute and subacute glomerulonephritis modified by adrenocorticotropin. *Proc Soc Exp Biol Med.* 1950;74(1):57-59.

18. Farnsworth EB. Studies on influence of adrenocorticotrophin in acute nephritis, in simple nephrosis and in nephrosis with azotemia. In: Mote JR, ed. *Proceedings of the First Clinical ACTH Conference*. Philadelphia: Blakiston Company; 1950:297-317.

19. Frankel M, Groen J, Hellinga G. Observations in a patient with chronic nephritis with nephrotic syndrome, during administration of corticotropic hormone. *J Clin Endocrinol Metab.* 1950;10(7):799-800.

20. Fujiwara T, Kikuchi N, Shibuya T, Kusakabe I, Aida M. An infantile case of nephrotic syndrome: successfully treated with intravenous administration of ACTH, and complicated with "recovery syndrome (Gomez)". *Tohoku J Exp Med.* 1962;75:319-331.

21. Greenman L, Weigand FA, Danowski TS. Therapy of the nephrotic syndrome: sodium restriction, dextran, and corticotropin (ACTH) alone or combined with nitrogen mustard. *AMA Am J Dis Child.* 1955;89(2):169-191.

22. Hecker H, Stevens RE. Nephrosis -- a long remission after ACTH. *R I Med J.* 1952;35(1):81-84.

23. Heidorn GH, Schemm FR, Layne JA. The varied patterns of water and sodium diuresis during corticotropin (ACTH) therapy of the nephrotic syndrome. *Am J Med Sci.* 1955;229(2):180-187.

24. Heidorn GH. The effect of corticotrophin (ACTH) on ammonia production in the nephrotic syndrome. *Am J Med Sci.* 1956;231(6):644-654.

25. Heidorn GH, Schemm FR. The clinical use of corticotropin (ACTH) and adrenal corticosteroids in the therapy of intractable edema. *Am J Med Sci.* 1955;229(6):621-631.

26. Heymann W, Spector S, Matthews LW, Shapiro DJ. Treatment of the nephrotic syndrome with corticotropin (ACTH) and cortisone: a four and one half year survey of results with short-term courses. *Am J Dis Child.* 1955;90(1):22-27.

27. Heymann W, Gilkey C, Salehar M. Effect of adrenocorticotropic hormone (ACTH) and cortisone on proteinuria and hematuria in the nephrotic syndrome. *Pediatrics.* 1955;15(1):49-53.

28. Hooft C, Herpol J. Aminoaciduria in the course of lipoid nephrosis in children; the influence of ACTH. *Acta Paediatr.* 1959;48(2):135-148.

29. Hooft C, Vermassen A, Herpol J. Reversible gluco-amino-phosphaturia in a child with lipoid nephrosis. *Helvetica Paediatrica Acta.* 1959;14(1):1-12.

30. Kohn JL, Gribetz D. Nephrotic syndrome of childhood. Comparison of results of current hormonal therapy with those achieved previously. *Am J Dis Child.* 1960;100(3):373-379.

31. Kramer B, Casden DD, Goldman H, Silverman SH. Effect of the adrenocorticotropic hormone (ACTH) on nephrosis in childhood. *Postgrad Med.* 1952;11(5):439-446.

32. Kramer B, Goldman H, Cason L. The treatment of the nonedematous nephrotic child with ACTH. *J Pediatr.* 1952;41(6):792-803.

33. Lange K, Slobody L, Strang R. Treatment of nephrotic syndrome with interrupted ACTH or oral cortisone therapy. *Proc Soc Exp Biol Med.* 1953;82(22):315-317.

34. Lange K, Slobody L, Strang R. Prolonged intermittent ACTH and cortisone therapy in the nephrotic syndrome; immunologic gasis and results. *Pediatrics.* 1955;15(2):156-168.

35. Lange K, Strang R, Slobody LB, Wenk EJ. The treatment of the nephrotic syndrome with steroids in children and adults. *AMA Arch Intern Med.* 1957;99(5):760-770.

36. Lange K, Wasserman E, Slobody LB. Prolonged intermittent steroid therapy for nephrosis in children and adults. *JAMA.* 1958;168(4):377-381.

37. Lauson HD, Forman CW, McNamara H, Mattar G, Barnett HL. Effect of corticotropin (ACTH) on glomerular permeability to albumin and on blood antidiuretic hormone concentration in children with the nephrotic syndrome. *AMA Am J Dis Child.* 1952;83(1):87-91.

38. Lauson HD, Forman CW, McNamara H, Mattar G, Barnett HL. The effect of corticotropin (ACTH) on glomerular permeability to albumin in children with the nephrotic syndrome. *J Clin Invest.* 1954;33(4):657-664.

39. Leyva FR, Sowers M. The use of ACTH and cortisone in the nephrotic syndrome in children. *Clin Proc Child Hosp DC.* 1953;9(12):262-265.

40. Luetscher JA, Jr., Deming QB, Johnson BB, Harvey J, Lew W, Poo LJ. Treatment of nephrosis with pituitary adrenocorticotrophin. *J Clin Invest.* 1951;12(2):1530-1541.

41. Mateer FM, Weigland FA, Greenman L, Weber J Jr, Kunkel GA, Danowski TS. Corticotropin (ACTH) therapy of nephrotic syndrome in children. I. Clinical results and effects on proteinuria in one hundred six instances. *J Dis Child.* 1957;93(6):591-603.

42. McCall MF, Ross A, Wolman B, Burns AD, Harpur EM, Goldbloom A. The nephrotic syndrome in children treated with A.C.T.H. and cortisone. *Arch Dis Child.* 1952;27(134):309-321.

43. McKean RM, Mack HC, Macy IG, Wiseman ME, Moyer EZ. Plasma protein patterns in a case of lipoid nephrosis; an electrophoretic study. *Harper Hosp Bull.* 1953;11(4):145-151.

44. Merrill AJ, Wilson J, Timerlake LF. Continuous therapy of nephrotic syndrome in children with corticotropin gel. *Arch Intern Med.* 1954;94(6):925-930.

45. Metcoff J, Rance CP, Kelsey WM, Nakasone N, Janeway CA. Adrenocorticotrophic hormone (ACTH) therapy of the nephrotic syndrome in children. *Pediatrics.* 1952;10(5):543-566.

46. Michaels L, Walters G. Increased haematuria in nephritis during cortisone and A.C.T.H. administration. *Arch Dis Child.* 1953;28(139):213-216.

47. Rance CP, Chute AL. Treatment of the nephrotic syndrome in children. *Can Med Assoc J.* 1955;73(12):959-964.

48. Rapoport M, McCrory WW, Michie AJ, et al. Effects of corticotrophin on children with nephrotic syndrome: clinical observations on 34 children; the effect of cortisone in 4. *AMA Am J Dis Child.* 1951;82(2):248-253.

49. Rapoport M, McCrory WW, Barbero G, Barnett HL, Forman CW. Effect of corticotropin (ACTH) on children with the nephrotic syndrome. *JAMA.* 1951;147(12):1101-1106.

50. Riley CM. Corticotropin and cortisone in management of the nephrotic syndrome in children. *JAMA.* 1952;150(13):1288-1291.

51. Riley CM, Davis RA, Fertig JW, Berger AP. Nephrosis of childhood: statistical evaluation of the effect of adrenocortical-active therapy. *J Chronic Dis.* 1956;3(6):640-650.

52. Rinvik R. Corticotropin in the treatment of the nephrotic syndrome. *J Oslo City Hosp.* 1952;2:149-152.

53. Schemm FR, Layne JA, Qualls EE. Adjuvants to hormonal therapy in the nephrotic syndrome. *Int Rec Med Gen Pract Clin.* 1957;170(1):11-26.

54. Schoolman LR. A case of nephrotic syndrome treated with ACTH. *Md State Med J.* 1956;5(10):622-628.

55. Shreeve WW, Hutchin ME, Harper HA, Miller CD, Doolan PD. Excretion of amino acids in nephrosis. *Proc Soc Exp Biol Med.* 1955;88(4):510-514.

56. Singh S. Nephrotic syndrome treated with ACTH. *Antiseptic.* 1952;49(1):54-57.

57. Soffer LJ, Elster SK, Hamerman DJ. Treatment of acute disseminated lupus erythematosus with corticotropin and cortisone. *AMA Arch Intern Med.* 1954;93(4):503-514.

58. SOSHEA JW, Farnsworth EB. Serum lipid analysis in the nephrotic syndrome under ACTH administration. *J Lab Clin Med.* 1951;38(3):414-419.

59. Stickler GB, McKenzie BF, Wakim KG, Burke EC. The effect of plasma transfusion and treatment with corticotropin on the electrophoretic patterns in serum and urine of children with the nephrotic syndrome. *J Lab Clin Med.* 1956;47(3):392-402.

60. West CD. Use of combined hormone and mechlorethamine (nitrogen mustard) therapy in lipoid nephrosis. *AMA J Dis Child.* 1958;95(5):498-515.
